# RNA-seq data of control and powdery mildew pathogen (*Golovinomyces orontii)* treated transcriptomes of *Helianthus niveus*

**DOI:** 10.1016/j.dib.2017.12.051

**Published:** 2018-01-04

**Authors:** Mulpuri Sujatha, Kandasamy Ulaganathan, Bhupatipalli Divya Bhanu, Prashant Kumar Soni

**Affiliations:** a ICAR- Indian Institute of Oilseeds Research, Rajendra nagar, Hyderabad 500030, Telangana, India; b Centre for Plant Molecular Biology, Osmania University, Hyderabad 500007, Telangana State, India

**Keywords:** *Golovinomyces orontii*, *Helianthus niveus*, Powdery mildew, RNA-seq, Transcriptome

## Abstract

Identification of genes expressed during the *Golovinomyces orontii* infection process in *Helianthus niveus* assumes importance for incorporation of resistance to powdery mildew in cultivated sunflower (*H. annuus* L.) from this donor species. RNA-seq analysis of control (uninfected) and infected samples of *H. niveus* resulted in identification of 231,754 transcripts. A total of 3726 transcripts were differentially expressed of which 205 were specifically expressed in control and 1961 in infected samples. Functional annotation of the differentially expressed transcripts showed significant upregulation of GRAS type transcription factor (TF) and plant specific GATA-type zinc finger TF in infected samples and the K-box, MADS box TF and WRKY family TF in control tissues. Gene ontology classification showed that genes involved in cell and cell part functioning, catalytic and metabolic processes were significantly and highly expressed. This is the first application of RNA-Seq for identification of key genes and pathways involved in powdery mildew infection process in a *Helianthus* species conferring resistance to the pathogen.

**Specifications Table**

**Table:** 

**Subject area**	*Biology*
**More specific subject area**	*Plant Molecular Biology*
**Type of data**	*Table, figures*
**How data was acquired**	*Illumina sequencing (Illumina-HiSeq system)*
**Data format**	*Filtered and analysed*
**Experimental factors**	*Comparison of H. niveus control and infected samples following infection with Golovinomyces orontii*
**Experimental features**	*RNA from control and infected samples subjected to RNA-Sequencing and transcriptome profiling*
**Data source location**	*Hyderabad, India*
**Data accessibility**	https://www.ncbi.nlm.nih.gov/sra/SRR3597501/

**Value of the data**

• Powdery mildew is a serious problem on sunflower (*Helianthus annuus* L.) in the tropics causing significant yield losses.
• *H. niveus,* a diploid annual and compatible species was identified as a reliable source of resistance to *Golovinomyces orontii.*
• The transcriptome data set generated for control and pathogen treated samples of *H. niveus* helps in identification of differentially expressed genes and pathways for a better understanding of the molecular mechanism of the defense response in *Helianthus* species to *G. orontii* infection.

## 1 Data

The dataset submitted to NCBI include the assembled transcriptome sequences of control and pathogen treated plants of *H. niveus* in Fasta format and the raw reads. Raw reads of both transcriptomes can be accessed with the following NCBI accession number: SRR3597501 (https://www.ncbi.nlm.nih.gov/sra/SRR3597501/).

**Fig. 1:**
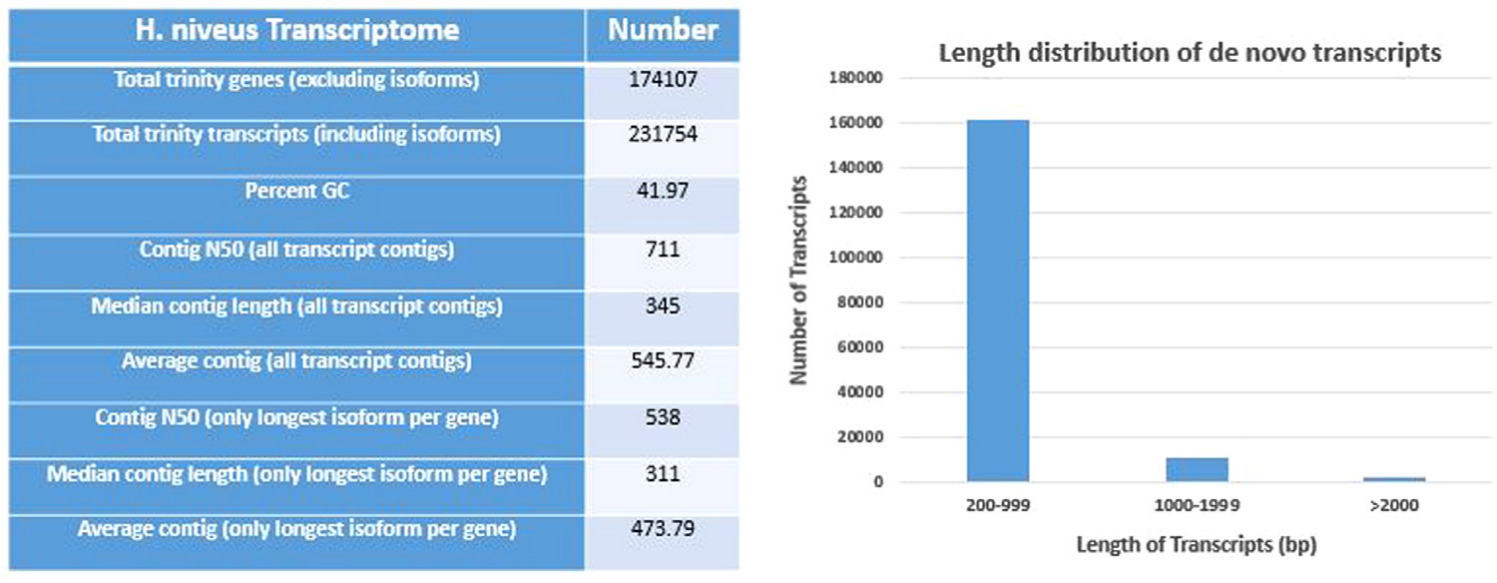
*Helianthus niveus* transcriptome statistics.

The summary of the transcriptomes is listed in Table 1. The transcriptome statistics and the length distribution of the *de novo* transcripts of *H. niveus* are presented in Fig. 1.

**Table 1.** Summary of *Helianthus niveus* (NIV1452) transcriptome.

**Feature**	***H. niveus*****1452 control**	***H. niveus*****1452_Pool**
NCBI Bio project ID	PRJNA320343	PRJNA320343
NCBI Bio sample ID	SAMN04932649	SAMN04932649
NCBI SRA accession number	SRR3597501	SRR3597501
NCBI transcriptome accession number	GEWS00000000	GEWS00000000
Sequence type	Illumina Hiseq	Illumina Hiseq
Total number of reads	83,17,546	13,65,44,538
Read length	100	100
No. of *de novo* transcripts	45,348	87,795

**Fig. 2:**
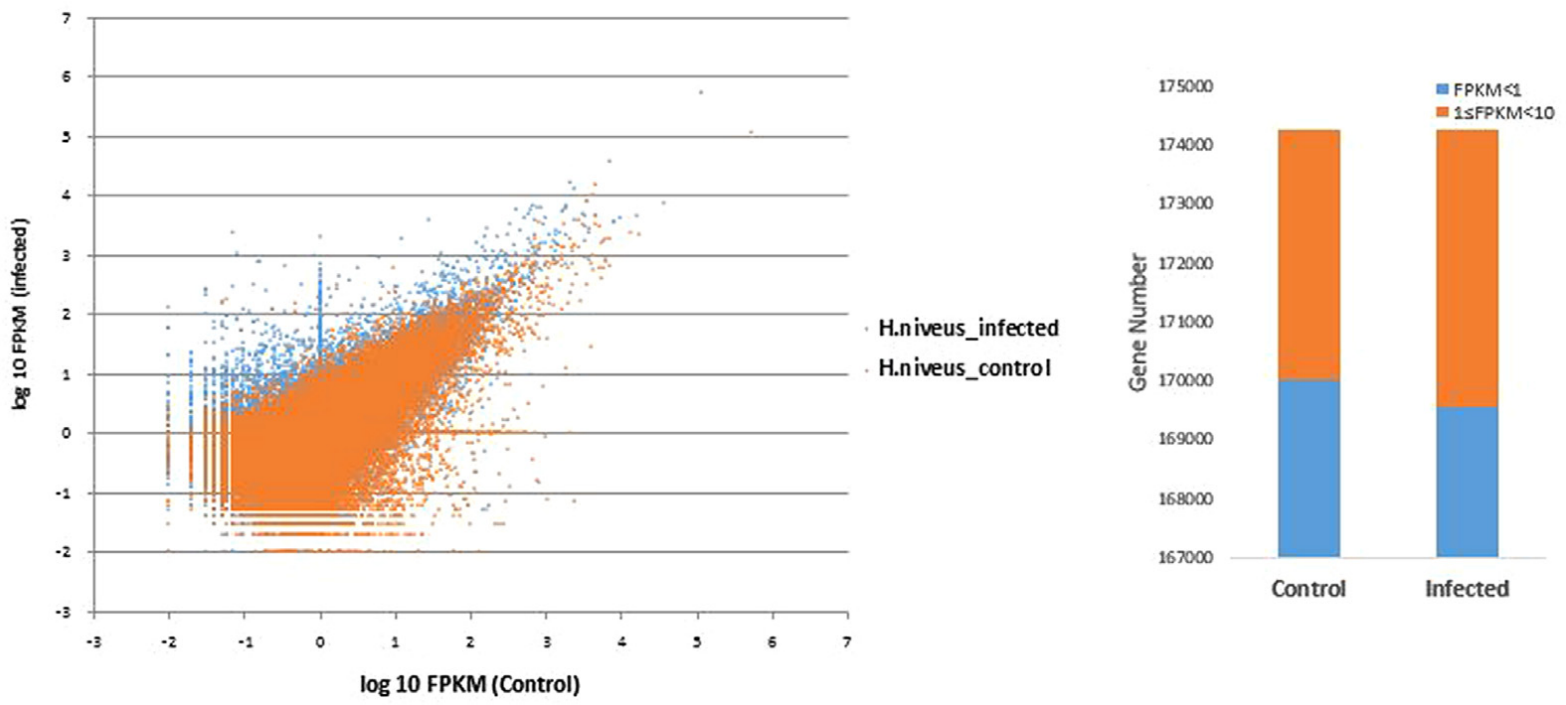
Expression values plotted as scatter plot showing common and differentially expressed genes and stacked column showing number of genes in FPKM ranges.

The transcript abundance in both the data sets was calculated in FPKM. The scatter plot of the normalized log 10 FPKM values clearly indicated that the most number of differentially expressed genes are from the infected sample. Both the control and infected samples showed minor difference in the number of genes having normalized FPKM values < 1 and 1 ≤ FPKM <10 (Fig. 2).

**Fig. 3:**
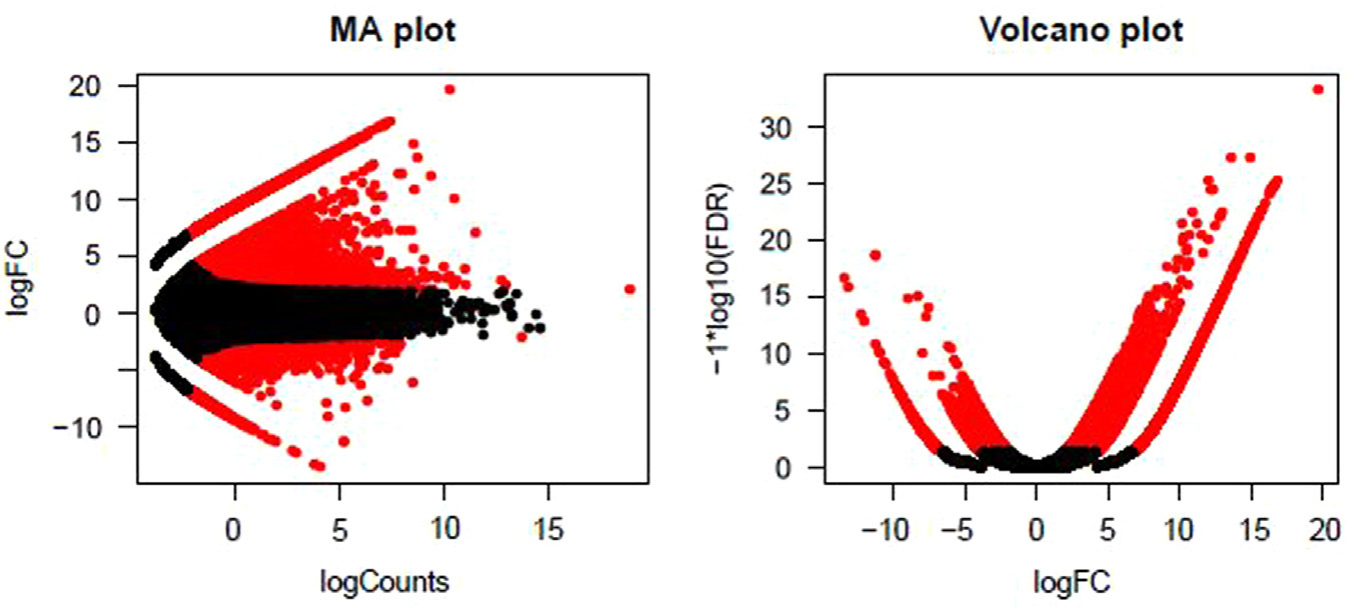
Transcript abundance based on expression profiles (a) MA plot - plots for each gene its log2 (fold change) between control and infected samples (*A*, Y axis) vs. its log2 (average expression) in control and infected samples (*M*, X axis). (b) Volcano plot comparing false discovery rate (-log10 FDR, Y axis) as a function of log2 (fold-change) between control and infected samples (log FC, X axis).

**Fig. 4:**
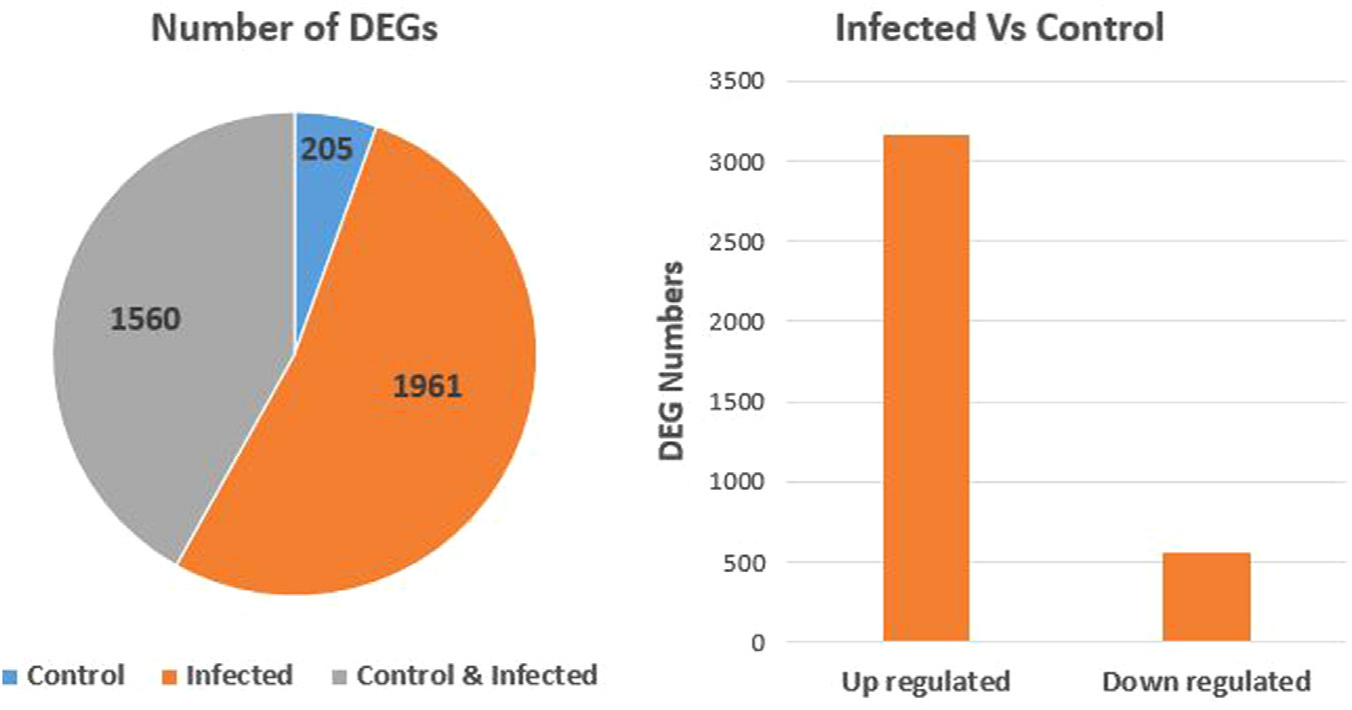
Summary of DEGs. A total of 3166 genes were up-regulated in the infected sample and 560 genes were down-regulated when compared to control.

The MA and volcano plots are shown in Fig. 3. The summary of the differentially expressed genes is represented in Fig. 4.

**Fig. 5:**
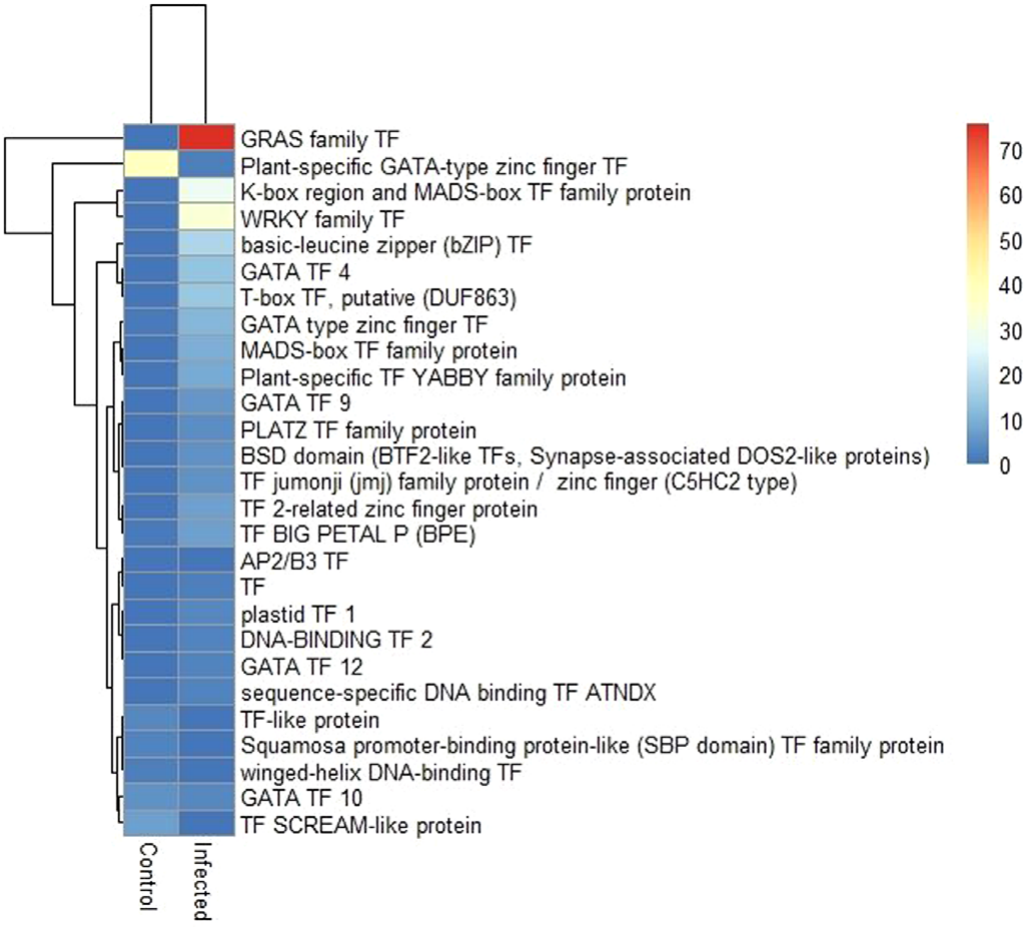
Heatmap showing the expression of transcription factors in control and pathogen treated *Helianthus niveus* leaves. GRAS family TF and Plant specific GATA-type zinc finger TF is highly expressed in infected sample compared to control while K-box and MADS box TF and WRKY family TF were highly expressed in control.

The functional annotation of differentially expressed genes showed many transcription factors and are shown in the heatmap (Fig. 5).

**Fig. 6:**
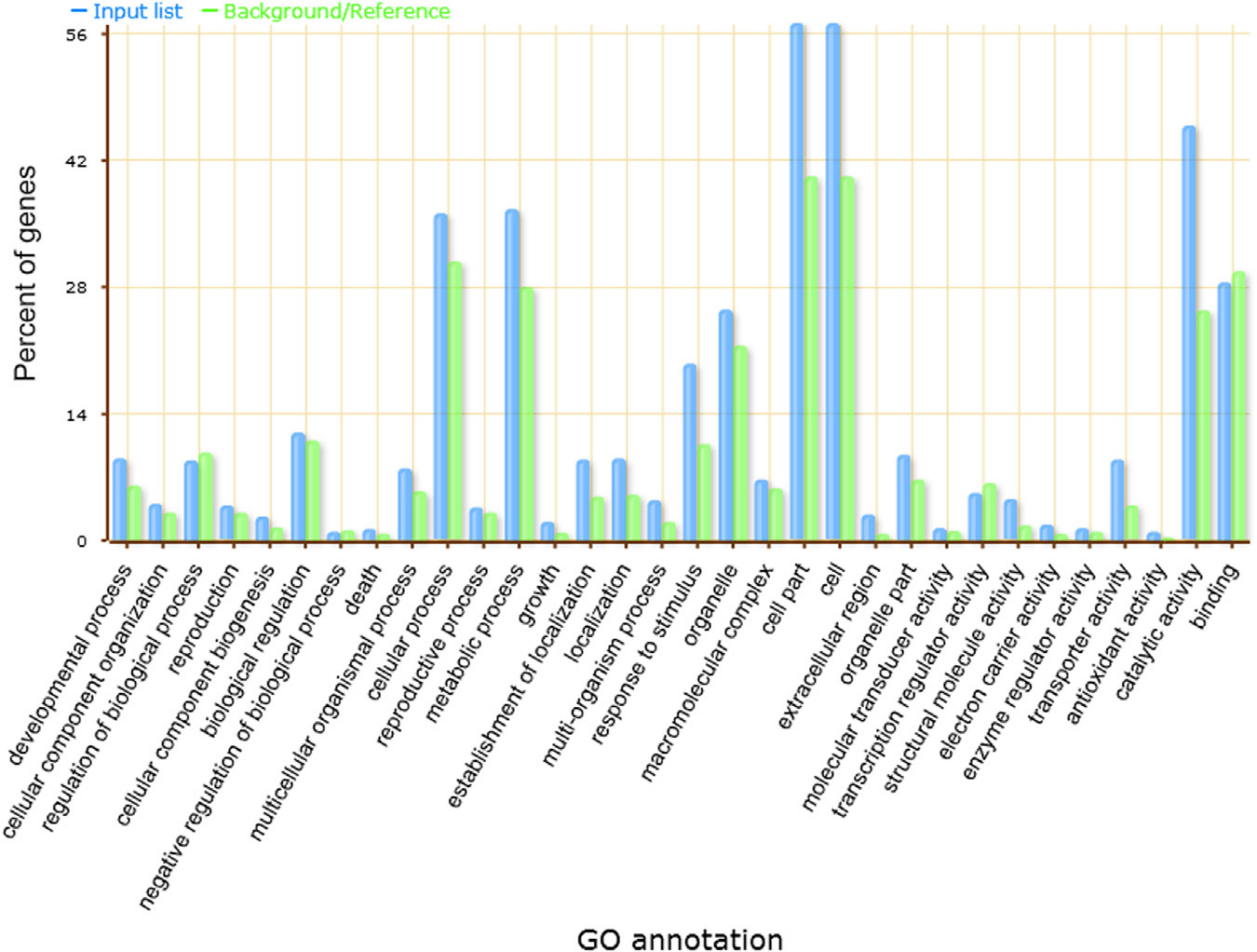
GO annotation classification of differentially expressed genes. in Fig. 6 shows that genes involved in cell and cell part functioning are highly differentially expressed followed by the genes involved in catalytic activity, cellular and metabolic processes. The cell and cell part fall in cellular component of gene ontology classes, catalytic activity is part of molecular function and cellular and metabolic process are part of biological process. This clearly indicates that the differentially expressed genes are part of all the three classes of gene ontology.

**Fig. S1:**
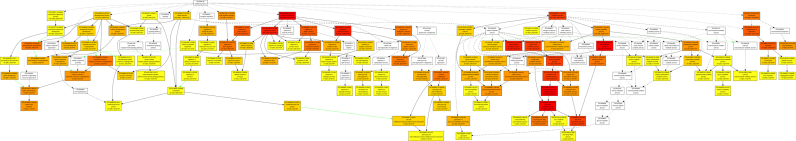
GO annotation-biological processes.

**Fig. S2:**
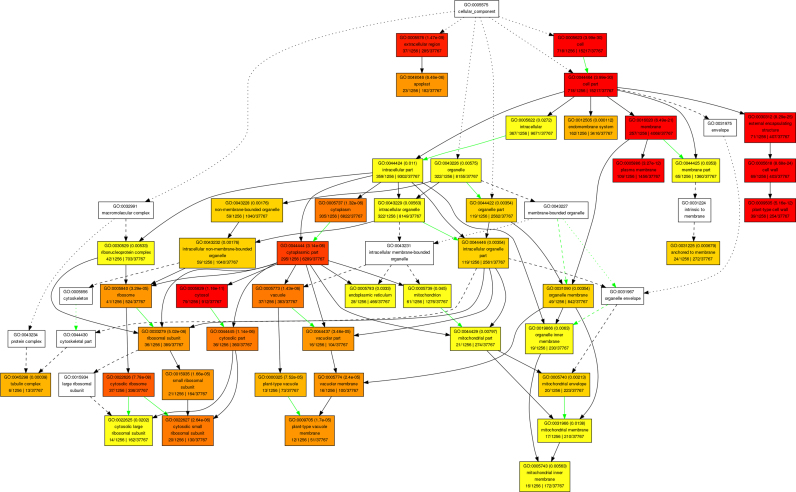
GO annotation-cellular components.

**Fig. S3:**
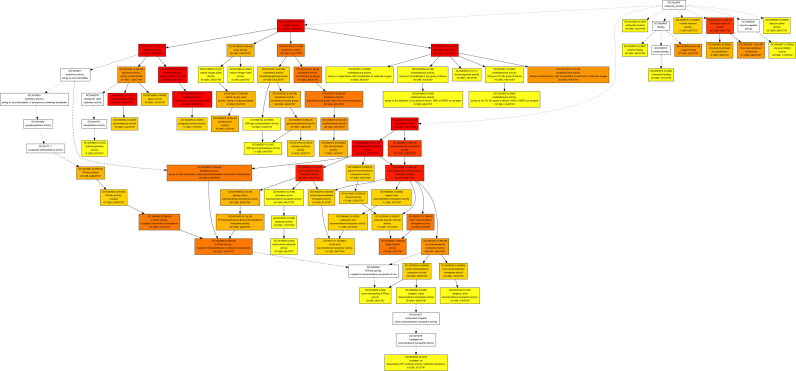
GO annotation-molecular functions Hope this information serves the purpose.

The gene ontology classification is presented in Fig. 6 and the supplementary figures (1, 2 and 3) represent each gene ontology class.

## 2 Data Interpretation

Plant-pathogen interactions involve a cascade of reactions in disease development. Plants have both resistance and defense genes which are activated through various signaling peptides. In this study, the leucine rich peptides (LRRs) which signal the activation of defense genes after contact with avirulence gene products of pathogens and the large gene family of WD40 proteins, were the top most highly regulated proteins in the infected sample when compared to the control. The structure and functions of these proteins have been extensively studied in plants suggesting a critical role of these repeating peptides in plant-pathogen interactions, plant cell physiology, stress and development [1]. The gene ontology graph showed differential and higher expression of the syntaxin-KNOLLE like protein which is involved specifically in cytokinetic vesicle fusion [2], Armadillo BTB protein 1 (ABAP1) that has a regulatory role and interacts with pre-replication complex (pre-RC) subunits [3] and the cysteine rich gibberellin regulated family proteins that are mainly involved in plant developmental regulation process [4] in the infected samples. Enzymes like aldehyde dehydrogenases (ALDHs) and O-acyl transferases were also highly expressed in the infected sample when compared to control. ALDHs are involved in plant growth, development, and stress responses while O-acyl transferases are membrane bound proteins [5].

The genes expressed highly in control (uninfected samples) include xyloglucan endotransglycosylase, uncharacterized protein and protein kinase. Before encountering the intracellular defense, a pathogen has to face the cell wall, which has an important role in plant defense. Xyloglucan endotransglycosylases catalyze transfer of a segment of one xyloglucan molecule allowing for molecular grafting between the polysaccharide molecules that subsequently change both the cell wall plasticity and architecture [6]. Protein kinases are major post-translational regulators of numerous cellular processes and are mainly involved in signaling pathways [7].

Earlier, differential gene expression studies are reported in sunflower for various traits but large-scale transcriptome data was used to identify genes in response to *Verticillium dahliae* infection [8]. In the present study, the transcriptome data set generated for control and pathogen treated samples of *H. niveus* provide a wealth of genomic information for a better understanding of the molecular mechanism of the defense response in this diploid annual *Helianthus* species to *G. orontii* infection. Further, the genomic resources in terms of the SSRs and SNPs that could be mined using the transcriptome data and the candidate genes identified serve as a prelude for transfer of the trait through marker assisted selection.

## 3 Experimental design, materials and methods

### 3.1 Plant material

The seeds of *H. niveus* (Accn No 1452) were soaked in water overnight. The seed coats were removed and the decoated seeds were plated in petri plates lined with moist filter paper. Following germination, seedlings were transferred to pots. When the plants were at the flowering stage (vulnerable stage for powdery mildew infection), pots were transferred to greenhouse (28 °C, 70% RH). Leaves were dusted with the powdery mildew conidia from infected leaves of the susceptible cultivated sunflower accession PS 2023B. Infected leaves were fixed at 0 (no infection), 24, 48 and 72 h post infection and subjected to transcriptome profiling.

### 3.2 Library preparation and sequencing

Leaf tissue (lamina of topmost leaf) was collected from control- and pathogen-treated (individual and pooled sample of 24, 48 and 72 hours post infection) plants and stored in “RNAlater” solution (Thermo Fisher Scientific) at -80 °C. RNA isolation was carried out using the RNeasy Plant kit (Qiagen). The leaf samples were ground to fine powder using liquid nitrogen in a mortar and pestle and subsequent isolation steps were as per the instructions provided in the RNeasy Plant Kit. The concentration and purity of the RNA was determined using a NanoDrop Spectrophotometer (Thermo Scientific - 1000). The integrity of the extracted RNA was analyzed on a Bioanalyzer (Agilent - 2100). Control and pathogen treated RNA samples with 7.9 to 8.2 RNA integrity numbers were used for library preparation.

Library preparation was performed using Illumina TruSeq RNA library protocol developed by Illumina Technologies (San Diego, CA). One µg of total RNA was subjected to PolyA purification of mRNA. Purified mRNA was fragmented for 8 min at elevated temperature (94 °C) in the presence of divalent cations and reverse transcribed with SuperScript III reverse transcriptase by priming with random hexamers. Second strand cDNA was synthesized in the presence of DNA polymerase I and RNaseH. The cDNA was cleaned up using HighPrep PCR reagent (MAGBIO, Cat# AC-60050). Illumina adapters were ligated to the cDNA molecules after end repair and addition of A base. SPRI (solid-phase reversible immobilization, Beckman Coulter) cleanup was performed after ligation. The library was amplified using 8 cycles of PCR for enrichment of adapter ligated fragments. The prepared library was quantified using Qubit and validated for quality by running an aliquot (1 µl) on High Sensitivity DNA Kit (Agilent) which showed expected fragment distribution in the range of ~250–500 bp. The effective sequencing insert size was ~130–380 bp; the inserts were flanked by adapters whose combined size was ~130 bp. Transcriptome sequencing was carried out with a Illumina-HiSeq system (Illumina, San Diego, CA) to obtain 80 million reads per sample.

### 3.3 De novo assembly

The raw paired-end reads were filtered for Illumina adapters/primers using Cutadapt [9] and subjected to *de novo* assembly using Trinity software [10], [11] with default K-mers = 25. Trinity combines three independent software modules: Inchworm, Chrysalis, and Butterfly, applied sequentially to process large volumes of reads. Inchworm assembles the data into the unique sequences of transcripts. Chrysalis clusters the Inchworm contigs into clusters and constructs complete de brujin graphs for each other. Butterfly then processes each graph independently to extract full-length splicing isoforms and to tease apart transcripts derived from paralogous genes. Zhao et al. [12] compared several *de novo* transcriptome assemblers and different assembly strategies, and found that Trinity was the best single K-mer assembler for transcriptome assembly. An assembled transcriptome (AS) was developed using the reads of both control and pathogen treated samples. Using bowtie2 [13], the assembled transcriptome was indexed and the reads were back aligned individually to the transcriptome to analyze the read composition of the assembly. A perl script in built in the trinity package was used to count the number of proper and improper read alignments.

### 3.4 Transcript expression or abundance

The abundances of transcripts generated by trinity were calculated using RSEM software. The typical two steps of preparing the reference followed by alignment of reads to the transcripts to estimate abundance from a run of RSEM was carried out using in built trinity perl script. Here, the reads of control and pathogen treated RNA were separately aligned to the transcriptome to get the transcript abundance of each data set individually. Normalized expression values of control and pathogen treated were generated separately as TPM values (transcripts per million reads) taking into account the transcript length, the number of reads mapped to the transcript and the total number of reads that mapped to any transcript. These TPM values of both control and pathogen treated samples were converted to matrix count file for giving as input to differential gene analysis.

### 3.5 Differential gene expression analysis

Differential gene expression (DGE) analysis is one of the most popular downstream analysis of RNA-seq data mainly because it gives clear variability between two or more datasets based on the expression values. Our interest was to identify the genes differentially expressed between control and pathogen infected transcriptomes of *H. niveus*. The edgeR package of R Bioconductor [14] taking a dispersion value of 0.1, P ≥ 0.001 and log fold change (logFC) as 2^(2) was used for DGE analysis. The differentially expressed genes between control and infected samples were estimated with a False Discovery Rate (FDR) value of at most 0.001 (colored red) and at least four-fold difference in expression values. The MA plot (where M=log ratios and A=mean values) and volcano plot were developed in which the MA plot takes log CPM on the X-axis and log FC on the Y axis whereas the volcano plot takes log FC on the X-axis and log FDR on the Y-axis.

### 3.6 Functional annotation, gene ontology and enrichment of differentially expressed genes

The differentially expressed genes were functionally annotated using blast2go [15], blastx [16] and AgBase programs [17]. The results were subjected for gene ontology enrichment using AgriGO [18].

### 3.7 Pathway mapping of differentially expressed transcripts

The differentially expressed transcripts in control and pathogen treated samples were mapped to biological pathways using a web-based Kyoto Encyclopedia of Genes and Genomes (KEGG) automatic annotation server (KAAS) by executing BlastX against the manually curated KEGG GENES (Kyoto Encyclopedia of Genes and Genomes) database. The result contains KO (KEGG Orthology) assignments and automatically generated KEGG pathways.
