# Supplementary material for: RNA-seq data of control and powdery mildew pathogen (Golovinomyces orontii) treated transcriptomes of Helianthus niveus
Source: Data Brief. 2018 Jan 4;17:210–7. doi: 10.1016/j.dib.2017.12.051 (PMC5988023; doi:10.1016/j.dib.2017.12.051)
Supplement: Supplementary file 1 — Supplementary material [file mmc1.doc]

**CONFLICT OF INTEREST DISCLOSURE**

I on behalf of all the authors have no conflicts of interest to declare

Sincerely,

M. Sujatha

(25-10-2017)
